# Evaluation of Tracheostomy Patients in Our Pediatric Intensive Care Unit: A Single-Center Study

**DOI:** 10.7759/cureus.66620

**Published:** 2024-08-11

**Authors:** Baris Kolbasi, Emine Senkal, Mustafa Taskesen

**Affiliations:** 1 Pediatric Health and Diseases, Dicle University Faculty of Medicine, Diyarbakır, TUR; 2 Pediatrics and Child Health, Health Education England, London, GBR

**Keywords:** paediatric respiratory, indication, complication, tracheostomy, paediatric intensive care

## Abstract

Objectives: A tracheostomy is a surgical procedure that can be performed on critically ill patients of all ages in intensive care units as indicated, and its use has been increasing in recent years. The most common indications are prolonged mechanical ventilation and upper airway obstruction. This study aimed to examine the indications for tracheostomy, assess the outcomes of patients who underwent the procedure, and identify the factors affecting these outcomes.

Material and method: A retrospective analysis of patients who underwent tracheostomy between 2013 and 2019 at Dicle University Faculty of Medicine Hospital Paediatric Intensive Care Unit (PICU). The patients' age, gender, distribution by age, primary diagnosis at admission to the intensive care unit, indication for tracheostomy, presence of additional disease, type of respiratory support before and after tracheostomy, development of complications (perioperative/postoperative), decannulation status, mortality, and discharge status were recorded.

Results: A total of 61 patients were enrolled into the study. The average age of the patients was 81.72 months (SD = 17.5), with the youngest being eight months old and the oldest being 203 months old. Of the 61 patients included in the study, 32 (52%) were male and 29 (48%) were female. The majority of patients (32 patients) were in the preschool age group (25-84 months). The primary diagnosis of 27 patients (44.3%) who underwent tracheostomy was neuromuscular diseases, and the most common indication for tracheostomy was prolonged intubation (24 patients, 39.3%). Concomitant chronic diseases were present in 54 patients (88.5%). Patients received mechanical ventilation support for an average of 47.34 days before tracheostomy. Early tracheostomy (0-21 days after initiation of mechanical ventilation) was performed on 14 patients, and late tracheostomy (21 days and later) was performed on 47 patients. Complications developed in nine patients (14.8%) in the perioperative period and in 19 patients (31.1%) in the postoperative period, while no complications developed in 39 patients (63.9%). Six patients (9.8%) were decannulated. Furthermore, 28 patients (45.9%) died. No tracheostomy-related mortality was documented.

Conclusion: Despite most patients being of preschool age, having prolonged intubation prior to tracheostomy, and having accompanying chronic illnesses, tracheostomy remains a frequently used procedure in paediatric intensive care units due to its low complication rates, making it an essential intervention that facilitates discharge from paediatric intensive care.

## Introduction

Child health is a critical area that shapes the future of public health. In contemporary discussions on child health, a holistic approach is taken to address various issues such as vitamin deficiencies, nutritional disorders, insidious heavy metal poisoning, and substance abuse. Apart from preventable child deaths, another area that paediatricians have increasingly focused on is paediatric intensive care and its complications. One of the complex procedures in Paediatric Intensive Care Units (PICUs) is tracheostomy [1,2]. 

Tracheostomy is a procedure that has been applied since ancient times for medical purposes. It is a Latin word that describes the medical procedure of creating a new communication between the airway tract below the larynx and environment, for medical purposes [3]. Over the years, the indications for tracheostomy in children have evolved. Initially, infections such as epiglottitis and laryngotracheitis were among the primary indications for tracheostomy [4,5]. However, in recent years, following the increase in the efficacy of treatment methods used in intensive care units, tracheostomies are more frequently performed on ventilator-dependent patients who need prolonged mechanical ventilation. In the paediatric age group, the most common reasons for performing a tracheostomy are upper airway obstruction, prolonged need for positive pressure mechanical ventilation, and the need for pulmonary care [3,4]. 

In a retrospective study involving 42 patients, it was reported that tracheostomy was most frequently performed due to prolonged intubation (97%), and complications developed in 23% of the patients in the early period (within the first seven days postoperatively) and in 14% in the late period (after seven days postoperatively), with 14% of the cases being decannulated [6]. 

Our study aimed to investigate the demographic characteristics of patients with tracheostomy, the indications for the procedure, and the complications that developed during follow-up. We also analysed decannulation rates, the impact of tracheostomy on weaning from the ventilator, and the length of stay in the PICU for these patients. Additionally, we examined post-tracheostomy mortality rates to determine if these deaths were direct complications of the tracheostomy or its management.

## Materials and methods

This retrospective study included 61 paediatric patients who were treated at Dicle University Faculty of Medicine Hospital PICU between 2013 and 2019 and underwent tracheostomy during their PICU stay. The inclusion criteria were: all patients admitted to the PICU (our PICU age limit is 17 years old), excluding neonates (<28 days old). The study was approved by the Ethics Committee for Clinical Research at Dicle University with the decision number 2019/256 on 14.11.2019. 

A retrospective review was conducted on the medical records of the patients. The study data was updated by contacting the parents by telephone to obtain information about their post-discharge status. 

The data included the patients' age, gender, age, primary diagnosis, presence of chronic disease, indication for intensive care admission, indication for tracheostomy, timing of tracheostomy (early/late), duration of mechanical ventilation before and after tracheostomy, type of respiratory support before and after tracheostomy, presence or development of intra‐operative and postoperative complications, decannulation status, survival status 28 after tracheostomy, the postoperative day on which complications occurred, the location of complications, the postoperative day on which mechanical ventilation was discontinued, and the location where mortality occurred if it occurred. 

We used day 21 as a cut-off point to define early and late tracheostomy. Early tracheostomy was defined as tracheostomy performed within 21 days after intubation, while late tracheostomy was defined as tracheostomy performed after this period. The age distribution was classified as infant (1-24 months), preschool age (25-84 months), school age (85-120 months), and adolescent age (121-204 months). The indications for tracheostomy were classified as prolonged intubation, upper airway obstruction, craniofacial anomalies, neurological diseases, trauma, and vocal cord paralysis. The location of complications was categorized as home and hospital. 

The data obtained from the patients included in the study were analysed using SPSS for Windows v24.0.0 program (IBM Corp., Armonk, NY, USA). Chi-square, Mann-Whitney U test, and Student t-test were used for statistical analysis. Values of p < 0.05 were considered statistically significant. 

## Results

The study included a total of 61 patients. When comparing the gender distribution across age groups, it was found that 14 of the female patients and 18 of the male patients were in the preschool age group. Statistically, since p > 0.05 (test value 2.592, p = 0.459), no significant difference was found between gender and age distribution. The mean age of the patients was 81.72 ± 43.20 months, ranging from eight months to 203 months. The majority were in the preschool age group (25-84 months) (Table 1). In terms of age distribution, 32 patients were in the preschool age group, 14 patients were in the adolescent age group, 11 patients were in the school-age group, and four patients were infants. Statistically, since p > 0.05 (test value 12.40, p = 0.414), no significant difference was found between the tracheostomy indications and age distribution.

**Table 1:** Tracheostomy Patients by Age Group

Age group	Number	Percentage
Infant (2-24 months)	4	6.6
Preschool Age (25-84 months)	32	52.5
School Age (85-120 months)	11	18.0
Adolescent Age (121-204 months	14	23.0
Total	61	100.0

Comparing the day of PICU admission on which the tracheostomy was performed and the tracheostomy indication revealed a significant difference between the groups, as p < 0.05 (p = 0.018). The Mann-Whitney U test was used for pairwise comparisons of groups with significant differences. A significant difference was found in the timing of tracheostomy procedures performed for prolonged intubation versus upper airway obstruction, with a p-value of 0.002 (p < 0.05). This indicates that the day of PICU admission when tracheostomies were performed differs significantly between these two groups.

The most common reason for performing a tracheostomy was extended intubation, accounting for 39.3% of cases, followed by upper airway obstruction at 27.9%. Thirteen patients required a tracheostomy because of neurological diseases. Additionally, three patients needed a tracheostomy due to bilateral vocal cord paralysis, and three patients needed it because of trauma. It was observed that 27 patients (44.3%) had neuromuscular diseases as their primary diagnosis, whereas 22 patients (36%) had lung infections (Table 2). 

**Table 2:** Tracheostomy indications by age group

Tracheostomy Indication	Infant	Preschool	School	Adolescent	Total	% of Total
Prolonged Intubation	2	9	4	9	24	39.3%
Upper airway obstruction	2	12	3	0	17	27.9%
Neurological diseases	0	7	3	3	13	21.3%
Trauma	0	1	1	1	3	4.9%
Vocal cord paralysis	0	3	0	1	4	6.6%
Total	4	32	11	14	61	100%

When comparing the presence of chronic diseases and age distribution in patients, it was found that 54 patients had pre-existing chronic diseases. Among these 54 patients, 29 were in the preschool age group, 11 were in the adolescent age group, 10 were in the school-age group, and four were infants. Statistically, since p > 0.05 (test value 2.084, p = 0.555), no significant difference was found between the presence of chronic diseases and age distribution.

Among the patients who received a tracheostomy, the mean duration of intubation before tracheostomy was 47.34 days, time varied between one day to 231 days. Early tracheostomy (0-21 days) was performed on 14 patients, and late tracheostomy (21 days and later) was performed on 47 patients. On average, tracheostomy was performed 63.10 ± 60.95 days after admission to the PICU, with the earliest tracheostomy being performed one day after admission and the latest tracheostomy being performed 305 days after admission. 

Intraoperative complications developed in nine (14.8%) patients and postoperative complications developed in 19 (31.1%) patients, while no complications developed in 39 (63.9%) patients. Perioperative complications included cannula malposition (n=4), cannula dislodgement (n=2), subcutaneous emphysema (n=2), and minor bleeding (n=1). The most common postoperative complication was stomal granulation (n=6), followed by ostium stenosis, tracheal granuloma, tracheoesophageal fistula, and wound infection in decreasing order. In patients with stomal granulation, the granulation tissue was excised under local anaesthesia, and ostium dilation was performed in five patients with ostium stenosis. Two out of four patients who developed tracheal granuloma needed surgical intervention. Two patients developed tracheoesophageal fistula and subsequently had surgical intervention. Two patients developed wound infection and were treated with wound debridement and systemic antibiotic therapy. The average time for the development of complications in the postoperative period was 11.86 days, with the latest complication occurring 66 days after the surgery. No mortality occurred due to tracheostomy or tracheostomy-related complications in any of the patients (Table 3). 

**Table 3:** Tracheostomy complications

Complications	Number	Percentage
Intraoperative complications	9	14.8%
Cannula malposition	4	
Cannula dislodgement	2	
Subcutaneous emphysema	2	
Minor bleeding	1	
Postoperative complications	19	31.1%
Stomal granulation	6	
Ostium stenosis	5	
Tracheal granuloma	4	
Tracheoesophageal fistula	2	
Wound Infection	2	
Total	28	45.9%

After tracheostomy, 34 (55.7%) patients received mechanical ventilation support, while 27 (44.3%) were managed without the need for mechanical ventilation support. The average duration of mechanical ventilation support in the postoperative period was 15.67 days.

Six patients (9.8%) were decannulated. The tracheostomy indication in these decannulated patients was prolonged intubation in four, upper airway obstruction in one, and trauma in one. Comparing the distribution of tracheostomy indications based on decannulation status, it was found that among the six decannulated patients, four had prolonged intubation, one had upper airway obstruction, and one had trauma as the indication for tracheostomy. Statistically, since p > 0.05 (test value 5.284, p = 0.259), no significant difference was found between the tracheostomy indications and the decannulation status.

During the follow-up after tracheostomy, mortality developed in 28 (45.9%) of the 61 patients, with 26 patients dying in the hospital and two at home. While no tracheostomy-related mortality was observed, the overall mortality rate was found to be 45.9%. 

When comparing the age distribution of patients who died, it was found that a total of 28 patients died (26 in the hospital and two at home). Among the 26 patients who died in the hospital, 16 were in the preschool age group, five were in the adolescent age group, four were in the school-age group, and one was an infant. Statistically, since p > 0.05 (test value 3.584, p = 0.733), no significant difference was found between the age distribution and the place of death.

Comparing the distribution of deaths based on the primary diagnosis at the time of PICU admission, it was found that out of the 28 deaths, 14 were due to neuromuscular diseases, 10 due to lung diseases, two due to trauma, one due to infectious diseases, and one due to malignancy. Statistically, since p > 0.05 (test value 1.687, p = 0.891), no significant difference was found between the death outcomes and the distribution of primary diagnoses at the time of ICU admission.

## Discussion

Over the past few years, our intensive care unit has seen an increase in the number of patients, the need for mechanical ventilation, and longer ventilation durations. This has resulted in a growing number of tracheostomy procedures. The decision to perform a tracheostomy in paediatric patients is influenced by several factors, including pre-existing medical conditions, length of ventilation and severity of airway obstruction.

Tracheostomy has several advantages, including improved patient comfort, more effective clearance of the airway, reduced airway resistance, increased mobilization and comfort of the patient, and no impediment to speech and swallowing, especially in infants, allowing for feeding [3,4]. Due to these advantages, it is thought that the duration of stay in mechanical ventilation and intensive care units can be shortened. However, the risk of complications is higher in the paediatric age group compared to the adult age group. These complications are known to develop secondary to both the surgical procedure and tracheostomy care [5].

In our single-center study, 52.5% of tracheostomy patients were in the preschool age group (25-84 months), with a total of 32 patients. This differs from findings by Sarıca et al., who reported that 50% of tracheostomy patients were below 12 months, and Özmen et al., where 23% of patients were under 12 months old [6,7]. We also found that in a comprehensive retrospective research study conducted by Donnelly et al., which analysed paediatric patients who had tracheostomy, it was found that the ratio of children aged under 12 months decreased significantly from 90% in the period between 1971 and 1980 to 26% in the period between 1981 and 1990 [8]. Fifty-five percent of our cases were male patients. Male predominance was also reported in similar series in the literature [6,7]. 

Our findings showed that prolonged intubation was the most common indication for tracheostomy, accounting for 39.3% (n=24) of cases. This aligns with other research, such as the study by Süslü et al., which found prolonged intubation as the primary indication (84.9%). The primary reasons for prolonged intubation in their study were respiratory failure (45.3%), neuromuscular diseases (20.8%), and the postoperative period following major surgery (15.1%) [9]. In the 17-year study by Mahadevan et al., which investigated paediatric tracheostomy indications in a tertiary hospital in New Zealand from 1987 to 2003, 70% of the patients required tracheostomy due to upper airway obstruction, primarily caused by craniofacial dysmorphism [10]. In a five-year study by Butnaru et al. including 46 patients who underwent tracheostomy, the indications for tracheostomy were upper airway obstruction and prolonged intubation at nearly equal rates (43%-57%) [11]. Overall, the recent studies generally identify prolonged intubation as the most common indication for tracheostomy.

The study conducted by Hsu et al. demonstrated that patients who underwent tracheostomy after 21 days had a higher mortality rate and a longer weaning period. They suggested that a prolonged period of intubation might damage the local barrier and bronchial hygiene, hence increasing the likelihood of bacterial colonisation. Consequently, they proposed that there is no advantage to postponing tracheostomy [12]. The timing of tracheostomy varies in paediatric intensive care units, with a shorter duration for patients with upper airway obstruction and a longer duration for patients with neurological diseases and prolonged intubation [13]. In our study, tracheostomy was performed on average 63.10 (1-305) days after admission to the PICU. Early tracheostomy was performed on 14 (23%) patients, while late tracheostomy was performed on 47 (77%) patients, with our results being consistent with the literature. The reason for the high rate of late tracheostomies and the latest tracheostomy being performed approximately 10 months after admission was due to the families being indecisive about the procedure and not having a positive outlook on tracheostomy. 

In the literature, the most common early complications are reported as pneumomediastinum and pneumothorax, with early complication rates ranging from 5-49%. The most common late complication is tracheal stenosis, with late complication rates ranging from 7-63%. The total complication rate related to tracheostomy varies between 18-63% [9,14,15]. In the study by Sarica et al., the early complication rate was 23%, and the late complication rate was 14%, while in the study by Atmaca et al., the early complication rate was 13%, and the late complication rate was 16.7% [6,15]. In our study, the intraoperative complication rate was 14.8% (n=9), and the postoperative complication rate was 31.1% (n=19). Overall, no complications developed in 63.9% (n=39) of the patients. Differences among studies are believed to arise from variations in how complications are defined and reported across different studies. 

In the study by Zia et al., it was reported that approximately 80% of patients who underwent tracheostomy were successfully weaned off mechanical ventilation [16]. In our study, 27 (44.3%) of 61 patients were followed up without the need for mechanical ventilation support. We believe that the higher number of tracheostomies performed due to prolonged intubation contributed to our lower weaning rates from mechanical ventilation. As Sachdev et al. mentioned in their study, early tracheostomies can facilitate earlier discharge from the PICU, leading to improved resource utilization and significant cost savings [17].

In the paediatric age group, decannulation rates vary between 7-78%, with the highest decannulation rates observed in patients with upper airway obstruction [10,16,17]. In the study by Trey et al., it was reported that the decannulation success rate (74.7%) in patients who underwent tracheostomy due to upper airway obstruction was significantly higher compared to patients who underwent tracheostomy due to prolonged intubation (52.5%) [18]. The different decannulation rates in the literature may be due to variations in tracheostomy indications and the patient groups in each study. In our study, only six (9.8%) of 61 patients were decannulated. The tracheostomy indication for the six decannulated patients was prolonged intubation in four, upper airway obstruction in one, and trauma in one. The low decannulation rate in our patients may be related to the primary diagnoses of our patients being predominantly neuromuscular diseases and lung disease. 

In the literature, the mortality rate due to the tracheostomy procedure varies between 0.5-16%, while this rate increases to 28% when comorbidity-related mortality is included. The overall mortality rate has been reported between 19-59% in various series, with direct tracheostomy-related mortality ranging from 0-7% [13,14,19]. We believe that the significant differences in mortality rates are due to variations in follow-up periods and patient groups among studies. In our study, 28 (45.9%) of the 61 patients died, with 26 patients dying in the hospital and two at home. No direct tracheostomy-related mortality was observed, while the overall mortality rate was found to be 45.9%. The high overall mortality rate in our study is believed to be attributed to the severity of the primary diseases and comorbid conditions in our patient population, as well as the late timing of tracheostomies. Specifically, 14 deaths were associated with neuromuscular diseases and 10 with chronic lung diseases. 

The primary limitation of our study is that it is a single-center retrospective study with a limited number of patients from different subgroups, making it difficult to perform a comprehensive evaluation. 

## Conclusions

When performed appropriately under intensive care conditions, tracheostomy is a procedure with low mortality and morbidity rates that can be safely applied, facilitating weaning from mechanical ventilation and discharge of patients requiring lifelong ventilation. This was also reflected in our study. The most important factor determining the course of tracheostomized patients is the indication for tracheostomy. The most common indications for tracheostomy are prolonged intubation, upper airway obstruction, and neurological diseases, and less frequently, vocal cord paralysis and trauma. While there is no definitive time for tracheostomy, each patient should be evaluated individually, and patients with a need for mechanical ventilation lasting longer than three weeks should be considered for tracheostomy, and this period should not exceed four weeks. Considering earlier tracheostomy in appropriate paediatric patients to potentially improve weaning rates from mechanical ventilation and reduce the length of PICU stay.

It is also recommended that parents be thoroughly informed about tracheostomy and encouraged towards the procedure, and provided with education to ensure they can adequately care for their children at home after clinical stabilization. Given the limited number of paediatric intensive care units and bed capacity in our country, tracheostomy is considered a significant and beneficial option to reduce the chronic patient burden. 
